# Effects of neurofeedback versus methylphenidate for the treatment of attention-deficit/hyperactivity disorder protocol for a systematic review and meta-analysis of head-to-head trials

**DOI:** 10.1097/MD.0000000000012623

**Published:** 2018-09-28

**Authors:** Lixia Yan, Junhua Zhang, Yang Yuan, Samuele Cortese

**Affiliations:** a School of Education, Soochow University, Soochow; b School of Education, Jiangsu Key Laboratory for Big Data of Psychology and Cognitive Science, Yancheng Teachers University; c Department of Paediatrics, Yancheng traditional Chinese medicine hospital, Yancheng, China; d Center for Innovation in Mental Health, Academic Unit of Psychology; e Clinical and Experimental Sciences (CNS and Psychiatry), Faculty of Medicine, University of Southampton; f Solent NHS Trust, Southampton, UK; g New York University Child Study Center, New York, NY; h Division of Psychiatry and Applied Psychology, School of Medicine, University of Nottingham, Nottingham, UK.

**Keywords:** ADHD, meta-analysis, methylphenidate, neurofeedback, systematic review

## Abstract

**Introduction:**

Attention-deficit/hyperactivity disorder (ADHD) is developmental disorder characterized by inattention and/or hyperactivity/impulsivity. Psychostimulants, including methylphenidate (MPH), are recommended as a first-line pharmacological intervention, whereas neurofeedback (NF) has been proposed as a nonpharmacological option. The comparative effects of MPH and NF need further exploration. We will conduct a systematic review and meta-analysis of head-to-head randomized controlled trials (RCTs) comparing the efficacy and/or tolerability of MPH and NF in children/adolescents and adults with ADHD.

**Method and Analysis:**

We will include published as well as unpublished data. Two investigators will independently search PubMed, OVID, ERIC, Web of Science, ClinialTrials.gov, and a set of Chinese databases, including CNKI, CQVIP, and WanFang for head-to-head RCTs comparing MPH and NF. Experts will be contacted for unpublished data. The primary outcome will be the efficacy on ADHD core symptoms, measured by the change in the severity of ADHD symptoms, from baseline to endpoint and, if available, at follow-up (at any available time point). Secondary outcomes will be: dropouts for any reasons; efficacy on neuropsychological measures (working memory, inattention, and inhibition). We will conduct subgroup analyses to assess the impact of the following variables: age; type of NF; language of publication; comorbidities. Additionally, we will carry out meta-regression analyses to investigate the effect of sponsorship, year of publication, duration of intervention, and age of participants. Sensitivity analyses will be conducted to test the robustness of the findings. Risk of bias of individual studies will be assessed using the Cochrane risk of bias tool. Analyses will be performed using Comprehensive Meta-Analysis Software.

**Ethics and Dissemination:**

No ethical issues are foreseen. Results from this study will be published in a peer-reviewed journal and presented at relevant national and international conferences.

**Trials registration number:**

PROSPERO CRD42018090256.

## 1 Introduction

Attention-deficit/hyperactivity disorder (ADHD) is one of the most common neurodevelopmental disorders, ^[1–3]^ affecting about 5% of schoolaged children and around 2.5% of adults worldwide.^[4,5]^ ADHD not only has an important impact on virtually every aspect of a individual's physical and mental health, daily functioning, and academic/occupational performance in the short and long run, but also entails a huge economic burden to patients, families, and broader society.^[6–8]^ Currently, several pharmacological and nonpharmacological options, such as behavioral interventions/parenting skills, dietetic changes, and cognitive training, have been proposed for the treatment of ADHD. ^[9–11]^ Psychostimulants, including methylphenidate (MPH), are recommended as a first-line pharmacological option in several guidelines for the treatment of ADHD.^[12–17]^ MPH, the most commonly used psychostimulant in many countries, inhibits the reuptake of dopamine and norepinephrine, increasing dopaminergic and noradrenergic activity in the prefrontal cortex, which may contribute its efficacy and effectiveness in ADHD.^[18,19]^ Despite evidence from short-term randomized controlled trials (RCTs) pointing to large effect size (ES) (among the largest not only in psychiatry but also in general medicine) for MPH (teacher-reported ADHD symptom ratings [SMD –0.83; 95% confidence interval, CI: –0.96, –0.70], general behavior [SMD –0.68; 95% CI: –0.78, –0.60], and quality of life [SMD 0.61; 95% CI: 0.48–0.80]),^[20]^ there are concerns over its tolerability and mixed evidence on its long-term effects.^[18]^ Storebo et al stated that MPH may improve symptoms of ADHD but is associated with a relatively high risk of nonserious adverse events.^[21,22]^

Therefore, alternative nonpharmacological options directly targeting the pathophysiology of ADHD are currently being actively investigated. Among these, neurofeedback (NF) has been proposed by a number of research groups as an effective and safe option for ADHD.^[23,24]^ NF is a process of operant conditioning, which aims at improving self-regulation of brain activity.^[25,26]^ Meta-analytic evidence on the efficacy of NF for ADHD is currently mixed. Arns et al, 2014,^[27]^ pooled 15 studies (including 476 subjects from 11 prospective controlled studies and 718 subjects from 4 pre-post-test design studies) and concluded that NF treatment for ADHD can be considered “efficacious and specific,” with a large ES (0.8097 and 0.6862, respectively) for inattention and impulsivity and a medium ES (0.3962) for hyperactivity. By contrast, Cortese et al of the European ADHD Guidelines Group (EAGG), 2016,after pooling 13 RCTs (including 520 participants with ADHD), found that, although ratings from unblinded assessors show significant effects of NF in reducing ADHD core symptoms, ratings from probably blinded assessors fail to support NF as an effective treatment for ADHD core symptoms.^[28]^ Overall, we are not aware of any evidence suggesting that NF outperforms placebo-NF for ADHD.

Evidence on the comparative efficacy/effectiveness and tolerability of MPH and NF needs further investigation. Catala-Lopez et al, 2017 did a comprehensive network meta-analysis including, among other treatments, MPH and NF. MPH emerged as more efficacious than NF on ADHD symptoms and global functioning.^[29]^ However, the meta-analysis by Catala-Lopez et al, 2017, did not focused on the effects of MPH and NF on subdomains of ADHD separately (i.e., inattention and hyperactivity-impulsivity). This is of relevance given that previous studies have shown that inattention and hyperactivity/ impulsivity symptoms may have different degrees of sensitivity to different treatments.^[27]^ Additionally, Catala-Lopez et al, 2017,^[30]^ chose to use a dichotomous outcome (i.e., proportion of patients who displayed improvements in the symptoms of ADHD or global functioning on standardized rating scales), which may be less informative compared to continuous outcomes.

Furthermore, when considering the comparison between MPH and NF, a key aspect relates to sustained effects. A meta-analysis by Van Doren et al, 2018, focused on sustained effects (defined by these authors as follow-up at 2–12 months) of NF in ADHD. They found that, compared to nonactive control treatments, NF had significantly more durable treatment effects for at least 6 months following treatment, although the authors concluded that more studies are needed for a properly powered comparison of follow-up effects between NF and active treatments.^[31]^ Indeed, this meta-analysis could not inform on the sustained effect of NF and MPH directly because it combined MPH with other active treatments including attention training, cognitive training, physical activity training, and self-management.

Finally, another aspect that deserves further investigation relates to the comparative efficacy of MPH and NF on neuropsychological measures, such as working memory or sustained attention. This is of relevance because executive dysfunctions, albeit far from being universal in ADHD, affect a sizable portion of individuals with ADHD and impact on their academic and global functioning.^[32]^

Therefore, a number of questions still need to be answered in relation to the comparative efficacy and tolerability of MPH and NF.

## 2 Objectives

Our study aimed to fill these gaps by means of a systematic review and meta-analysis of head-to-head RCTs comparing the effects (at trial end point and, if available, at follow-up) in terms of efficacy of MPH and NF on separate ADHD core symptoms (inattention and hyperactivity/impulsivity), using continuous measures as outcome. We will also explore the feasibility of assessing the comparative effects on neuropsychological variables. We also will assess the comparative tolerability of MPH and NF.

## 3 Methods

Methods for this systematic review/meta-analysis have been developed according to recommendations from the Preferred Reporting Items for Systematic Reviews and Meta-Analyses.^[33]^

### 3.1 Eligibility Criteria

#### 3.1.1 Population

Individuals (children/adolescents [<18 years] and or adults [≥18 years]) with a categorical diagnosis of ADHD according to the DSM (III, III-R, IV, IV-TR or 5) or hyperkinetic disorder as per the ICD-10 or previous ICD versions or above cut point on any validated ADHD measure.^[34,35]^

#### 3.1.2 Intervention(s)

We will include trials comparing NF and MPH. Both fixed-dose and flexible-dose designs (in relation to the MPH regime) will be allowed. Studies assessing the efficacy of multimodal treatments including the combination of NF plus other treatments will be excluded.

#### 3.1.3 Comparator(s)/control

Studies including a nonactive comparator will be retained if they include at least 2 other active arms, that is, MPH and NF.

#### 3.1.4 Types of outcome(s)

The primary outcome will be the efficacy (as a continuous outcome) on the severity of ADHD core symptoms (at the end of the study and, if available, at follow-up, filled out by parents, teachers, patients or clinician(s). We will perform an analysis focusing on the total ADHD score, that is, inattentive plus hyperactive/impulsive symptoms, and another analysis focusing on ADHD subdomains, that is, analyzing separately inattention and hyperactivity/impulsivity. Validated ADHD rating scales that we will consider eligible for the measurement of the outcomes are reported in Table 1.^[36]^ As in previous studies,^[37]^ we will conduct separate analyses for measures rated by clinicians, parents, teachers, and patients (self). Secondary outcomes will be the number of dropouts for any reasons at the end of the intervention (and, if available, at follow-up) and neuropsychological laboratory-based measures of working memory (e.g., The visual spatial working memory task,^[38]^ attention (e.g., Test of Variables of Attention,^[39,40]^ Attention Endurance Test (d2)^[41]^), and inhibition (e.g., Integrated Visual and Auditory Continuous Performance Test).^[42]^

**Table 1:**
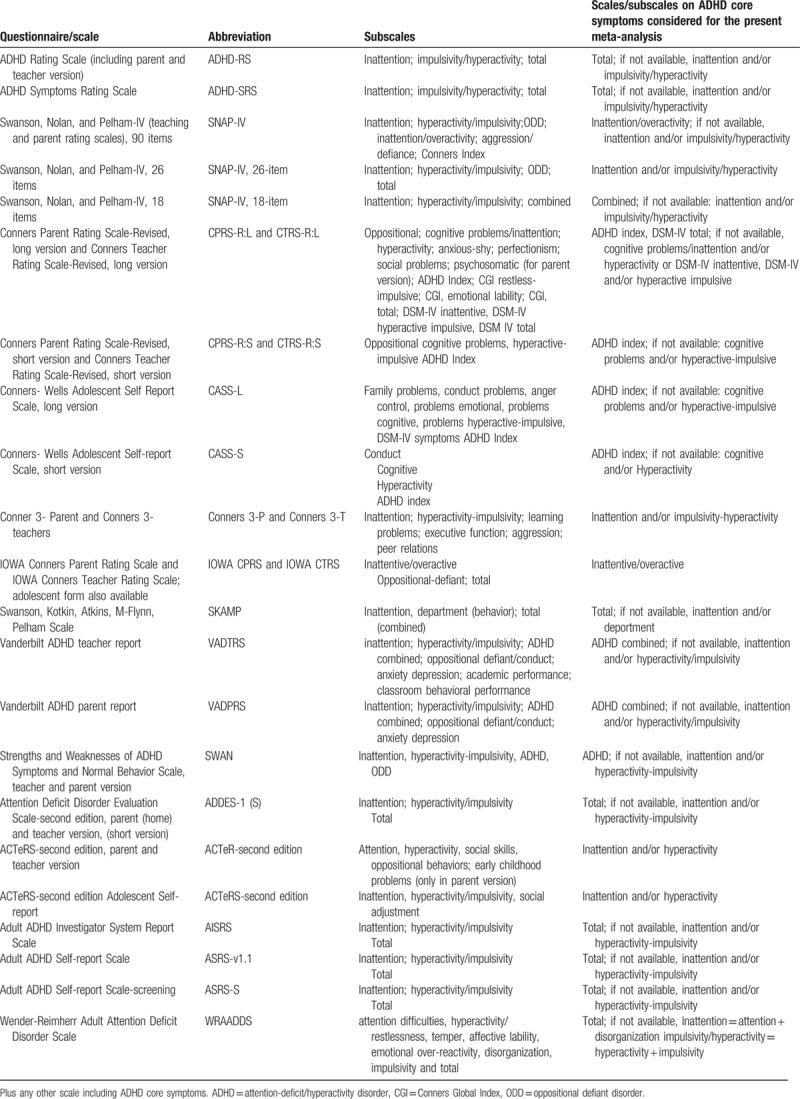
Scales/subscales for ADHD considered for possible inclusion.

Timing: the primary analysis will focus on endpoint- baseline changes; secondary analyses will focus on data, when available, at a follow-up (time point closest to 12 months after treatment as in Van Doren et al's, 2018, and other studies^[31,43]^, or longer follow-up, if available).

#### 3.1.5 Types of Study

RCTs will be included, regardless the level of blinding. Parallel-group RCTs, cross-over trials, and, if available, cluster trials will be considered. For cross-over studies, owing to concerns on possible “carry-over” effects,^[44]^ we will use data from the precross-over phase, whenever this is reported in the study report. When data for the precross-over phase are not reported, we will contact study authors to obtain them.

### 3.2 Search strategy

We will include published and unpublished studies pertinent to our criteria. An electronic literature search will be conducted independently by 2 authors. The following electronic databases will be searched with no language, date, and type of document restrictions: PubMed, OVID, ERIC, and Web of Science (including Science Citation Index Expanded (SCI-EXPANDED). Chinese databases, including China National Knowledge Infrastructure (CNKI), CQVIP, and WanFang data, will also be searched with the translated searching strategy. We will also search Clincaltrials.gov, clinicaltrialsregister.eu, and osf.io for additional reports not published in peer-reviewed journals.

The search terms/syntax in PubMed will be as follows:

(ADHD OR adhd OR attention deficit disorder with hyperactivity OR minimal brain disorder OR syndrome hyperkinetic OR hyperkinetic syndrome OR hyperactivity disorder OR hyperactive child syndrome OR childhood hyperkinetic syndrome OR attention deficit hyperactivity disorders OR attention deficit hyperactivity disorder OR adhd attention deficit hyperactivity disorder OR addh OR overactive child syndrome OR attention deficit hyperkinetic disorder OR hyperkinetic disorder OR attention deficit disorder hyperactivity OR attention deficit disorder hyperactivity OR child attention deficit disorder OR hyperkinetic syndromes OR syndromes hyperkinetic OR hyperkinetic syndrome childhood) AND (neurofeedback OR neuro feedback OR “EEG biofeedback” OR neuro therapy OR neurotherapy OR SCP OR “slow cortical potentials”) AND (Methylphenidate∗ OR methylphenidate∗ OR Ritalin OR ritalin)

The search strategy/syntax will be adapted for each database. The references of all selected studies will be hand searched for other published reports and citations of unpublished studies. Finally, we will contact experts in the field to query about any completed study not yet published.

### 3.3 Data extraction

Studies identified through electronic and manual searches will be listed with citation, titles, and abstracts in EndNote; duplicates will be excluded using the EndNote function “remove duplicates.” The eligibility for inclusion process will be conducted in 2 separate stages:

1. Two authors will independently screen title and abstracts of all nonduplicated articles and will exclude those not pertinent to the criterion. A final list will be agreed with discrepancies resolved by consensus between the 2 authors. When consensus is not reached, a third senior author will act as arbitrator. If any doubt about inclusion exists, the article will proceed to the next stage.
2. The full-text version of the articles passing stage 1 screening will be downloaded and assessed for eligibility by two authors, independently. We will provide a complete list of articles excluded at the full-text screening stage, with reasons for exclusion. Discrepancies will be resolved by consensus between the 2 authors and, if needed, a third senior author will act as arbitrator. Data from multiple reports of the same study will be linked together. Where required, we will contact the corresponding author to inquire about study eligibility.

One reviewer will input outcome data from studies included in previous systematic reviews into Excel. This will be independently cross-checked by another reviewer. The following data will be collected from each included study:

- Study details: first author/study ID, year(s) of study or publication, location (country or continent), setting, diagnostic criteria, funding/sponsor (industry or academic);
- Participants details, including sex distribution, mean and range of age, presence, and type of co-morbid (neuro) psychiatric conditions, mean (and SD) IQ, numbers in each group, and a number of dropouts for side effects in both groups.
- Interventions details, including mean and maximum doses of MPH, type of NF, the duration of interventions, and whether forced dose or optimized treatment with MPH; time(s) of outcome measurement
- Outcomes: mean, SD, or percentage in both groups at pre-test, post-test, and follow-up (any time point reported).
- Effect size, statistical power, and the original researcher’ hypothesis for each study.
- Information as to whether participants in the NF studies learn to regulate the feedback.

### 3.4 Risk of bias (quality) assessment

Risk of bias will be assessed for each included study using the Cochrane Collaboration risk of bias tool, as a reference.^[45]^ The risk of bias domains include selection bias, performance bias, detection bias, attrition bias, and other bias. Two independent review authors will assess the risk of bias in selected studies. The degree of agreement between the two independent raters will be reported. Any disagreement will be resolved through discussion and in consultation with the principal investigators. Where necessary (i.e., unclear information for the published report), the authors of the studies will be contacted for further information. As in Cortese et al,^[46]^ the overall rating of risk of bias for each study will be the lowest rating for any of the criteria (e.g., if any domain is scored high risk of bias, the study will be considered high risk of bias).

### 3.5 Data synthesis

Meta-analyses will be performed by means of Comprehensive Meta-Analysis Software (CMA) (http://www.meta-analysis.com/index.php) using the option “standardized by post score SD.” Additionally, we will use the appropriate function in CMA, to combine outcomes within study from the same subjects (https://www.meta-analysis.com/downloads/Multiple%20outcomes.pdf). Heterogeneity will be assessed and measured with Cochran's Q and *I*^2^ statistics, which estimates the percentage of variation among ES that can be attributed to heterogeneity.^[47]^*I*^2^ >0 indicates that the degree of heterogeneity is greater than would be expected by chance. Clinically significant values will be indicated by SMD >0.4.^[48]^

### 3.6 Subgroup and meta-regression analyses

We will explore the feasibility of conducting subgroup analyses to assess the impact of the following variables: age of population; the language in which studies were published; comorbidities. Additionally, we will carry out meta-regression analyses to investigate the effect of sponsorship, year of publication, duration of intervention, age of participants.

### 3.7 Publication bias

Publication bias will be assessed via funnel plots and Eggers test.^[49]^

### 3.8 Sensitivity analyses

We will perform the following sensitivity analyses by: removing studies rated at overall high risk of bias; excluding studies with small sample size trials (at least 30 children per arm); excluding studies where the diagnosis was not made according to standardized DSM/ICD criteria; excluding unpublished data; removing studies on nonstandard NF (i.e., theta/beta EEG ratio, sensorimotor rhythm, and slow cortical potentials) as per Arns et al, 2014.^[26]^ We will also explore the feasibility of conducting sensitivity analyses by:(1) excluding studies not using the Conners’ scale; (2) excluding studies without teachers’ rating.

### 3.9 Ethics and dissemination

This systematic review and meta-analysis will not undertake any primary data collection, so no ethical approval is required. The findings of this study will be published in a peer-reviewed journal.

## 4 Conclusion

This is a comprehensive meta-analysis of head-to-head trials of MPH versus NF using published and unpublished data. ADHD subdomains as well as neuropsychological measures will be considered separately. Comparative effects of MPH and NF on ADHD will be assessed not only at study endpoint but also at follow-up. A possible limitation is the inclusion of different rating scales to assess the core symptoms of ADHD. However, we will select only validated scales that measure exclusively the same triad of symptoms, that is, inattention, hyperactivity, and impulsivity.

## Author contributions

LY and JZ conceived the study and drafted the protocol. SC supervised the study design and edited the first draft of the protocol. SC and JZ designed the search strategy. LY and YY reviewed and commented on the protocol in PROSPERO. All authors (JZ, LY, YY, SC) contributed to the development of inclusion and exclusion criteria. All authors (JZ, LY, YY, SC) read, contributed, and approved the final manuscript.

**Conceptualization:** Lixia Yan, Junhua Zhang, Samuele Cortese.

**Data curation:** Junhua Zhang, Yang Yuan.

**Investigation:** Yang Yuan.

**Methodology:** Junhua Zhang, Yang Yuan.

**Supervision:** Samuele Cortese.

**Writing – original draft:** Lixia Yan.

**Writing – review & editing:** Samuele Cortese.

Lixia Yan orcid: 0000-0002-9578-2265

## References

[1] Barnett R Attention deficit hyperactivity disorder. Lancet (London, England) 2016;387:737. 10.1016/s0140-6736(16)00332-9 26913307

[2] Danckaerts M Sonuga-Barke EJ Banaschewski T The quality of life of children with attention deficit/hyperactivity disorder: a systematic review. Eur Child Adolesc Psychiatry 2010;19:83–105. 19633992 10.1007/s00787-009-0046-3 PMC3128746

[3] Biederman J Faraone SV Spencer TJ Functional impairments in adults with self-reports of diagnosed ADHD: A controlled study of 1001 adults in the community. The Journal of clinical psychiatry 2006;67:524–40. 16669717 10.4088/jcp.v67n0403

[4] Polanczyk GV Salum GA Sugaya LS Annual research review: A meta-analysis of the worldwide prevalence of mental disorders in children and adolescents. Journal of child psychology and psychiatry, and allied disciplines 2015;56:345–65. 10.1111/jcpp.12381 25649325

[5] Simon V Czobor P Balint S Prevalence and correlates of adult attention-deficit hyperactivity disorder: meta-analysis. The British journal of psychiatry: the journal of mental science 2009;194:204–11. 19252145 10.1192/bjp.bp.107.048827

[6] Cunill R Castells X Attention deficit hyperactivity disorder. Med Clin (Barc) 2015;144:370–5. 24787685 10.1016/j.medcli.2014.02.025

[7] Birnbaum HG Kessler RC Lowe SW Costs of attention deficit-hyperactivity disorder (ADHD) in the US: excess costs of persons with ADHD and their family members in 2000. Curr Med Res Opin 2005;21:195–206. 15801990 10.1185/030079904X20303

[8] Connolly JJ Glessner JT Elia J ADHD & pharmacotherapy: past, present and future: a review of the changing landscape of drug therapy for attention deficit hyperactivity disorder. Ther Innov Regul Sci 2015;49:632–42. 26366330 10.1177/2168479015599811 PMC4564067

[9] Sonuga-Barke EJS Brandeis D Cortese S Nonpharmacological interventions for ADHD: systematic review and meta-analyses of randomized controlled trials of dietary and psychological treatments. Am J Psychiatry 2013;170:275–89. 23360949 10.1176/appi.ajp.2012.12070991

[10] Cortese S Rosello-Miranda R Treatments for children and adolescents with attention deficit hyperactivity disorder: what is the evidence base to date? Rev Neurol 2017;64:S3–7. 28256680

[11] Richardson M Moore DA Gwernan-Jones R Non-pharmacological interventions for attention-deficit/hyperactivity disorder (ADHD) delivered in school settings: systematic reviews of quantitative and qualitative research. Health Technol Assess 2015;19:1–470. 10.3310/hta19450 PMC4780980 26129788

[12] NICE. Attention defificit hyperactivity disorder:diagnosis and management. In. National Clinical Practice Guideline Number 87. 14 March 2018 ed. London: National Institute for Clinical Excellence; 2018.

[13] Bolea-Alamanac B Nutt DJ Adamou M Evidence-based guidelines for the pharmacological management of attention deficit hyperactivity disorder: update on recommendations from the British Association for Psychopharmacology. J Psychopharmacol 2014;28:179–203. 24526134 10.1177/0269881113519509

[14] Wolraich M Earls M Feldman HM Subcommittee on Attention-Deficit/Hyperactivity Disorder; Steering Committee on Quality Improvement and Management. ADHD: clinical practice guideline for the diagnosis, evaluation, and treatment of attention-deficit/hyperactivity disorder in children and adolescents. Pediatrics 2011;128:1007–22. 22003063 10.1542/peds.2011-2654 PMC4500647

[15] Kooij SJ Bejerot S Blackwell A European consensus statement on diagnosis and treatment of adult ADHD: The European Network Adult ADHD. BMC Psychiatry 2010;10:67. 20815868 10.1186/1471-244X-10-67 PMC2942810

[16] Pliszka S AACAP Work Group on Quality Issues. Practice parameter for the assessment and treatment of children and adolescents with attention-deficit/hyperactivity disorder. J Am Acad Child Adolesc Psychiatry 2007;46:894–921. 17581453 10.1097/chi.0b013e318054e724

[17] CADDRA. Canadian ADHD Practice Guidelines. Available at: https://www.caddra.ca/canadian-adhd-practice-guidelines/ Accessed May 15, 2018.

[18] Myer NM Boland JR Faraone SV Pharmacogenetics predictors of methylphenidate efficacy in childhood ADHD. Mol Psychiatry 2017. 10.1038/mp.2017.234 PMC7039663 29230023

[19] Reddy DS Current pharmacotherapy of attention deficit hyperactivity disorder. Drugs Today (Barc) 2013;49:647–65. 24191257 10.1358/dot.2013.49.10.2008996

[20] Banaschewski T Buitelaar J Chui CS Methylphenidate for ADHD in children and adolescents: throwing the baby out with the bathwater. Evid Based Ment Health 2016;19:97–9. 27935807 10.1136/eb-2016-102461 PMC10699535

[21] Storebo OJ Krogh HB Ramstad E Methylphenidate for attention-deficit/hyperactivity disorder in children and adolescents: Cochrane systematic review with meta-analyses and trial sequential analyses of randomised clinical trials. BMJ 2015;351:h5203. 26608309 10.1136/bmj.h5203 PMC4659414

[22] Storebo OJ Pedersen N Ramstad E Methylphenidate for attention deficit hyperactivity disorder (ADHD) in children and adolescents - assessment of adverse events in non-randomised studies. Cochrane Database Syst Rev 2018;5:CD012069. 29744873 10.1002/14651858.CD012069.pub2 PMC6494554

[23] Duric NS Assmus J Elgen IB Self-reported efficacy of neurofeedback treatment in a clinical randomized controlled study of ADHD children and adolescents. Neuropsychiatr Dis Treat 2014;10:1645–54. 25214789 10.2147/NDT.S66466 PMC4159126

[24] Flisiak-Antonijczuk H Adamowska S Chladzinska-Kiejna S Treatment of ADHD: comparison of EEG-biofeedback and methylphenidate. Arch Psychiatry Psychother 2015;17:31–8.

[25] Strehl U What learning theories can teach us in designing neurofeedback treatments. Front Hum Neurosci 2014;8:894. 25414659 10.3389/fnhum.2014.00894 PMC4222234

[26] Arns M Heinrich H Strehl U Evaluation of neurofeedback in ADHD: the long and winding road. Biol Psychol 2014;95:108–15. 24321363 10.1016/j.biopsycho.2013.11.013

[27] Arns M de Ridder S Strehl U Efficacy of neurofeedback treatment in ADHD: the effects on inattention, impulsivity and hyperactivity: a meta-analysis. Clin EEG Neurosci 2009;40:180–9. 19715181 10.1177/155005940904000311

[28] Cortese S Ferrin M Brandeis D Neurofeedback for attention-deficit/hyperactivity disorder: meta-analysis of clinical and neuropsychological outcomes from randomized controlled trials. J Am Acad Child Adolesc Psychiatry 2016;55:444–55. 27238063 10.1016/j.jaac.2016.03.007

[29] Catala-Lopez F Hutton B Nunez-Beltran A The pharmacological and non-pharmacological treatment of attention deficit hyperactivity disorder in children and adolescents: a systematic review with network meta-analyses of randomised trials. PLoS One 2017;12:e0180355. 28700715 10.1371/journal.pone.0180355 PMC5507500

[30] Altman DG Royston P The cost of dichotomising continuous variables. BMJ 2006;332:108. 10.1136/bmj.332.7549.1080 PMC1458573 16675816

[31] Van Doren J Arns M Heinrich H Sustained effects of neurofeedback in ADHD: a systematic review and meta-analysis. Eur Child Adolsc Psychiatry 2018. 10.1007/s00787-018-1121-4 PMC6404655 29445867

[32] Willcutt EG Doyle AE Nigg JT Validity of the executive function theory of attention-deficit/hyperactivity disorder: a meta-analytic review. Biol Psychiatry 2005;57:1336–46. 15950006 10.1016/j.biopsych.2005.02.006

[33] Liberati A Altman DG Tetzlaff J The PRISMA statement for reporting systematic reviews and meta-analyses of studies that evaluate healthcare interventions: explanation and elaboration. BMJ 2009;339:b2700. 19622552 10.1136/bmj.b2700 PMC2714672

[34] Cortese S Are concerns about DSM-5 ADHD criteria supported by empirical evidence? BMJ 2013;347:f7072. 24285173 10.1136/bmj.f7072

[35] Doernberg E Hollander E Neurodevelopmental disorders (ASD and ADHD): DSM-5, ICD-10, and ICD-11. CNS Spectr 2016;21:295–9. 27364515 10.1017/S1092852916000262

[36] ML W Vanderbilt ADHD Teacher Rating Scale (VADTRS) and the Vanderbilt ADHD Parent Rating Scale (VADPRS). 2003 Avaialable at: www.nichq.org.

[37] Sonuga-Barke EJ Brandeis D Cortese S Nonpharmacological interventions for ADHD: systematic review and meta-analyses of randomized controlled trials of dietary and psychological treatments. Am J Psychiatry 2013;170:275–89. 23360949 10.1176/appi.ajp.2012.12070991

[38] Westerberg H Hirvikoski T Forssberg H Visuo-spatial working memory span: a sensitive measure of cognitive deficits in children with ADHD. Child Neuropsychol 2004;10:155–61. 15590494 10.1080/09297040409609806

[39] Greenberg LM An objective-measure of methylphenidate response—clinical use of the MCA. Psychopharmacol Bull 1987;23:279–82. 3615775

[40] Fuchs T Birbaumer N Lutzenberger W Neurofeedback treatment for attention-deficit/hyperactivity disorder in children: a comparison with methylphenidate. Appl Psychophysiol Biofeedback 2003;28:1–2. 12737092 10.1023/a:1022353731579

[41] Brickenkamp R Test d2, Aufmerksamkeits-Belastungs-Test Göttingen: Hogrefe; 1994.

[42] Moreno-Garcia I Meneres-Sancho S Camacho-Vara de Rey C A randomized controlled trial to examine the posttreatment efficacy of neurofeedback, behavior therapy, and pharmacology on ADHD measures. J Atten Disord 2017;1087054717693371-1087054717693371. 10.1177/1087054717693371 29254414

[43] Steiner NJ Frenette EC Rene KM In-school neurofeedback training for ADHD: sustained improvements from a randomized control trial. Pediatrics 2014;133:483–92. 24534402 10.1542/peds.2013-2059

[44] Curtin F Elbourne D Altman DG Meta-analysis combining parallel and cross-over clinical trials. III: the issue of carry-over. Stat Med 2002;21:2161–73. 12210631 10.1002/sim.1207

[45] Higgins JPT Green S Cochrane Handbook for Systematic Reviews of Interventions. Chichester: John Wiley and Sons; 2011.

[46] Cortese S Adamo N Mohr-Jensen C Comparative efficacy and tolerability of pharmacological interventions for attention-deficit/hyperactivity disorder in children, adolescents and adults: protocol for a systematic review and network meta-analysis. BMJ Open 2017;7:e013967. 10.1136/bmjopen-2016-013967 PMC5253538 28073796

[47] Borenstein M Hedges LV Higgins JPT Introduction to Meta-analysis. New Jersey: John Wiley & Sons, Ltd; 2009.

[48] Citrome L Quantifying clinical relevance. Innovations in clinical neuroscience 2014;11:26–30. PMC4140623 25152844

[49] Egger M Davey Smith G Schneider M Bias in meta-analysis detected by a simple, graphical test. BMJ 1997;315:629–34. 9310563 10.1136/bmj.315.7109.629 PMC2127453

